# Validation of basal metabolic rate equations in persons with innervated and denervated chronic spinal cord injury

**DOI:** 10.14814/phy2.16099

**Published:** 2024-06-14

**Authors:** Ahmad M. Alazzam, Ashraf S. Gorgey

**Affiliations:** ^1^ Spinal Cord Injury and Disorders Center, Richmond VA Medical Center Richmond Virginia USA; ^2^ Physical Medicine and Rehabilitation, School of Medicine Virginia Commonwealth University Richmond Virginia USA

**Keywords:** basal metabolic rate, indirect calorimetry, prediction equations, spinal cord injury

## Abstract

Basal metabolic rate (BMR) measurement is time consuming and requires specialized equipment. Prediction equations allow clinicians and researchers to estimate BMR; however, their accuracy may vary across individuals with chronic spinal cord injury (SCI). The objective of this study was to investigate the validity of SCI‐specific equations as well as able‐bodied (AB) prediction equations in individuals with upper motor neuron (UMN), lower motor neuron (LMN), and females with SCI. Twenty‐six men and women with chronic SCI (*n* = 12 innervated males, *n* = 6 innervated females, *n* = 8 denervated males) participated in this cross‐sectional study. BMR values were measured by indirect calorimetry. Body composition (dual‐energy X‐ray absorptiometry and anthropometrics) assessment was conducted. AB‐prediction equations [Cunningham, Nelson, Owen, Harris and Benedict, Mifflin, Schofield, Henry] and SCI‐specific equations [Chun and Nightingale & Gorgey] were used to estimate and validate BMR. The accuracy of AB‐specific FFM equations in predicting BMR was evaluated using Bland–Altman plots, paired t‐tests, and error metrics. Measured BMR for innervated males, females, and denervated males was 1436 ± 213 kcal/day, 1290 ± 114, and 1597 ± 333 kcal/day, respectively. SCI‐specific equations by Chun et al., Nightingale & Gorgey, and AB‐specific FFM equations accurately predicted BMR for innervated males. For the denervated males, Model 4 equation by Nightingale & Gorgey was not different (*p* = 0.18), and Bland–Altman analyses showed negative mean bias but similar limits of agreement between measured and predicted BMR for the SCI‐specific equations and AB‐specific FFM equations. We demonstrated that SCI‐specific equations accurately predicted BMR for innervated males but underpredicted it for denervated males. The Model 4 equation by Nightingale & Gorgey accurately estimated BMR in females with SCI. Findings from the current study will help to determine caloric needs in different sub‐groups of SCI.

## 1 INTRODUCTION

Following spinal cord injury (SCI), numerous changes in body composition and metabolism occur due to skeletal muscle paralysis below the level of injury, increased fat mass (FM), and reductions in fat‐free mass (FFM) (Gorgey & Gater, 2011; Spungen et al., 2003). Individuals who sustain a SCI often live a sedentary lifestyle, which has been shown to increase adiposity followed by a significant reduction in metabolically active lean body mass (LBM) that is comprised of muscle and bone below the level of injury (Castro et al., 1999; Gorgey et al., 2014; Gorgey & Dudley, 2007; Jones et al., 1998). Further reduction of LBM below the level of injury, impaired sympathetic nervous system activity, and low levels of physical activity contributes to altered metabolic activity and neurogenic obesity (Farkas & Gater, 2018; Gater Jr et al., 2021). Neurogenic obesity is characterized by a premature and disproportionate increase in body weight and adiposity in individuals with chronic SCI as compared to age‐matched, able‐bodied individuals (Farkas & Gater, 2018; Gater Jr et al., 2021). Additionally, across SCI literature, it has been found that the prevalence of neurogenic obesity varies from 82% to 85% among individuals with SCI, while 53%–60% was noted in overweight and obese persons with SCI (Nash et al., 2018, 2019). This subsequently predisposes persons with SCI to a wide range of health‐related comorbidities (Gill et al., 2020; Rajan et al., 2008).

Reductions in metabolically active lean tissue and impaired sympathetic nervous system activity also results in a decrease in basal metabolic rate (BMR) (Nightingale & Gorgey, 2018; Spungen et al., 1993). Additionally, FFM is more metabolically active compared to FM; therefore, reductions in FFM and increases in FM are closely related to decreases in BMR (Bauman et al., 2004; Gorgey et al., 2014; Spungen et al., 2003). The variability associated with body weight can be attributed to imbalances between energy intake and energy expenditure. A positive energy balance in persons with SCI increases the risk of obesity and is associated with negative metabolic sequelae, including glucose intolerance (Bauman, Adkins, et al., 1999; Bauman & Spungen, 1994), insulin resistance (Karlsson, 1999), hyperlipidemia (Bauman et al., 1992; Nash et al., 2016), and cardiovascular disease (Bauman, Spungen, et al., 1999; Krum et al., 1992). In contrast, a dramatic reduction in caloric intake could potentially result in malnutrition, reduction in physical activity, and fatigue that may affect activities of daily living (Wong et al., 2012). BMR accounts for 75%–80% of the total daily energy expenditure (TDEE) in persons with SCI and refers to the minimum daily energy requirements to maintain whole body homeostasis to support essential functions including breathing, circulation, nutrient processing, and cell production (Gorgey et al., 2015). Therefore, an accurate estimate of BMR is critical for determining total caloric needs for the SCI population.

Furthermore, individuals with paraplegia below T5/T6 may include thoracic, lumbar, and sacral injury levels that vary in somatic and sympathetic dysfunction and differ in neurological outcomes (Farkas et al., 2020). Lower motor neuron (LMN) injuries are more prevalent in individuals with SCI below T9/10 injury level (Doherty et al., 2002). Persons with LMN injury have been estimated to account for nearly 25% of the entire SCI population (Chandrasekaran et al., 2020). Additionally, a LMN injury results in extensive skeletal muscle atrophy, fibrillations in muscles, and infiltration of both intramyocellular fat and intramuscular fat (IMF) (Carraro et al., 2015; Chandrasekaran et al., 2020). A recent report demonstrated that muscle cross‐sectional area was lower by 38%–61% following a LMN compared to SCI in persons with upper motor neuron (UMN) injuries (Alazzam, Goldsmith, et al., 2023). Further, UMN injuries are due to disruption in the spinal cord, which can be characterized by loss of voluntary motor control, hyperreflexia, and increased muscle spasticity (Doherty et al., 2002). These substantial differences in muscle mass and quality between SCI individuals with LMN and UMN injuries are likely to influence BMR, which necessitates distinct considerations when estimating metabolic rate.

Indirect calorimetry is considered the gold standard for assessing BMR, which is based on the measurements of oxygen and carbon dioxide production and requires the use of expensive metabolic carts that are usually confined to research environments (Nevin, Mayr, et al., 2016). In order to achieve an accurate BMR measurements, participants/patients should be fasted for a minimum of 10–12 h and measured following an overnight stay in a dark, quiet, thermoneutral environment (22–26°C) (Compher et al., 2006). Measuring BMR via indirect calorimetry allows for accurate quantification of energy needs for individuals with SCI; however, in the absence of specific equipment for accurate assessment of BMR, prediction equations are often used in place to estimate BMR. Therefore, developing effective predictive equations is highly desirable to accurately estimate BMR in persons with SCI. This is likely to provide clinicians with a tool to accurately calculate BMR, a component of determining energy expenditure and caloric intake with the aim of preventing an unnecessary positive or negative energy balance individuals with SCI.

The BMR prediction equations serve as a surrogate for metabolic rate measurement and allow clinicians/dieticians to estimate energy needs for individuals with SCI. However, BMR is often estimated using prediction equations developed primarily for populations without SCI and incorporate separate equations based on sex (Table 1) (Harris & Benedict, 1918; Henry, 2005; Mifflin et al., 1990; Schofield, 1985). Several AB‐prediction equations have also incorporated FFM, a key determinant of BMR, which could accurately predict BMR rather than only using anthropometric variables (Cunningham, 1991; Nelson et al., 1992; Owen et al., 1987). A recent review by Farkas et al. evaluated the accuracy of numerous prediction equations reported in SCI and showed that several prediction equations derived from AB individuals overestimate energy expenditure for individuals with SCI, in addition to reporting that no studies examined metabolic rate exclusively in females (Farkas et al., 2019). Despite the availability of SCI‐specific prediction equations, clinicians and researchers often continue to use AB‐prediction equations due to their widespread familiarity and accessibility, which underscores the importance of their validating accuracy within the SCI population.

**TABLE 1:** Prediction equations for estimating basal metabolic rate.

Equation name/author(s)	Year	Sex	BMR/RMR prediction equations
AB‐specific equations for predicting BMR/RMR			
Cunningham (Cunningham et al., 1991)	1991	M/F	=370 + 21.6 × FFM
*Nelson et al. (Nelson et al., 1992)	1992	M/F	=25.80 × FFM + 4.04 × FM
*Owen et al. (Owen et al. 1987)	1987	M	=290 + 22.3 × FFM
		F	=334 + 19.7 × FFM
Harris & Benedict (Harris & Benedict, 1918)	1919	M	=66.4730 + (13.7516 × weight) + (5.0033 × height) – (6.7550 × age)
		F	=(1.8496 × height) + (9.5634 × weight) + 655.095 – (4.6756 × age)
*Mifflin et al. (Mifflin et al., 1990)	1990	M	=10 × weight + 6.25 × height – 5 × age + 5
		F	=10 × weight + 6.25 × height – 5 × age – 161
Schofield et al. (Schofield et al., 1985)	1985	M	=15.057 × weight + 692.2 (age, 18–30 yrs.), 11.472 × weight + 873.1 (age, 30–60 yrs.), 11.711 × weight + 587.7 (age, >60 yrs.)
		F	=14.818 × weight + 486.6 (age, 18–30 yrs.), 8.126 × weight + 845.6 (age, 30–60 yrs.), 9.082 × weight + 658.5 (age, >60 yrs.)
Henry (Henry, 2005)	2005	M	=16.0 × weight + 545.0 (age, 18–30 yrs.), 14.2 × weight + 593.0 (age, 30–60 yrs.), 13.5 × weight + 514.0 (age, >60 yrs.)
		F	=13.1 × weight + 558.0 (age, 18–30 yrs.), 9.74 × weight + 694.0 (age, 30–60 yrs.), 10.1 × weight + 569.0 (age, >60 yrs.)
SCI‐specific equations for predicting BMR			
Chun et al. (Chun et al., 2017)	2017	M/F	=24.5 × FFM + 244.4
Nightingale & Gorgey (Nightingale & Gorgey, 2018)	2018	M	=23.469 × FFM + 294.330 (Model 1)
		M	=23.995 × FFM + 6.189 × SAD +6.384 × TAD – 6.948 × TC + 275.211 (Model 2)
		M	=19.789 × FFM + 5.156 × weight + 8.090 × height – 15.301× CC –860.546 (Model 3)
		M	=13.202 × height + 11.329 × weight – 16.729 × TAD – 1185.445 (Model 4)

*Note*: the following table was adapted from Farkas et al. (2019). *Equations measured resting metabolic rate (RMR).

Abbreviations: AB, able‐bodied; BMR, basal metabolic rate; CC, calf circumference (cm); F, female; FFM, fat‐free mass (kg); M, male; RMR, resting metabolic rate; SAD, sagittal abdominal diameter (cm); SCI, spinal cord injury; TAD, transverse abdominal diameter (cm); TC, thigh circumference (cm).

SCI‐specific prediction equations have been developed by Chun et al. & Nightingale & Gorgey, which incorporate FFM via dual energy x‐ray absorptiometry (Chun et al., 2017; Nightingale & Gorgey, 2018). Previously, FFM was estimated using body weight in persons with SCI (Gorgey et al., 2012), which demonstrates the feasibility of estimating FFM without reliance on costly and specialized body composition assessment tools such as DXA scans. In addition, equations by Nightingale & Gorgey also included anthropometric measurements (circumferences and/or diameters) as using FFM as a single predictor might not be as sensitive in estimating BMR (Nightingale & Gorgey, 2018). Both equations were validated to predict BMR accurately in individuals with chronic motor complete SCI (Chun et al., 2017; Nightingale & Gorgey, 2018). However, recent findings have highlighted that these equations may underestimate energy requirements by 11% (Ma et al., 2022). However, the study did not account for the differences between BMR and resting metabolic rate (RMR) and other important factors similar to completeness of injury (complete vs. incomplete), gender (male vs. female), and neurological level of injury (innervated vs. denervated). Due to the heterogenous nature of the SCI population, further examination is warranted to assess the predictive performance of SCI‐specific equations in estimating BMR in different sub‐groups with SCI.

The primary aim of this study was to validate the commonly used AB‐prediction equations (Cunningham, 1991; Harris & Benedict, 1918; Henry, 2005; Mifflin et al., 1990; Nelson et al., 1992; Owen et al., 1987; Schofield, 1985) and SCI‐specific equations by Chun et al. and by Nightingale & Gorgey (Chun et al., 2017; Nightingale & Gorgey, 2018) in (1) innervated population with SCI, (2) denervated population with SCI, and (3) females with SCI. We hypothesized that prediction equations developed by Chun et al. and Nightingale & Gorgey for SCI populations would predict BMR more accurately within different sub‐groups of SCI compared to AB‐specific equations.

## 2 METHODS

### 2.1 Participants

Twenty‐six individuals with chronic (>1‐year post‐injury) SCI were recruited to participate in one of two clinical trials (NCT02660073 and NCT03345576). Recruitment for both clinical trials stated October 2015 and ended in March 2023. Study procedures were in accordance with the ethical standards of the 1964 Declaration of Helsinki. A neurological examination was performed per the International Standards for Neurological Classification of SCI (ISNCSCI) to determine the American Spinal Injury Association (ASIA) Impairment Scale (AIS) for each participant (Rupp et al., 2021). Physical characteristics of all participants are presented in Table 2. Exclusion criteria included cardiovascular disease, uncontrolled type II diabetes mellitus (HbA1c > 7.5), uncontrolled hypertension (resting blood pressure > 140/90 mmHg), insulin dependence, pressure sores stage 3 or greater, hematocrit above 50%, or severe urinary tract infection or symptoms. BMR for pre‐menopausal women was measured 10 days following the end of self‐reported menstruation cycle (Benton et al., 2020). Each participant signed an informed consent form that was approved by the Richmond VA Medical Center IRB. Additionally, denervation was confirmed by a lack of response to electrical stimulation of the knee extensor muscles. The data presented in this manuscript are cross‐sectional baseline data that was analyzed prior to any study intervention.

**TABLE 2:** Physical and clinical characteristics of denervated and innervated participants.

Characteristic	Total	Innervated males	Innervated females	Denervated males
	*N* = 26	*N* = 12	*N* = 6	*N* = 8
Sex	20 males; 6 females	12 males	6 females	8 males
Age (years)	44 ± 12	45 ± 13	43 ± 12	42 ± 10
Weight (kg)	72.6 ± 18.8	68.8 ± 18.9	69.5 ± 9.2	80.7 ± 23.1
Height (cm)	172.6 ± 9.7	177.9 ± 7.7	161.7 ± 7.8	172.8 ± 7
BMI (kg/m^2^)	24.8 ± 6.0	21.7 ± 5.6	26.6 ± 3.0	28.2 ± 6.5
Body fat (%)	35.0 ± 11.1	27.4 ± 8.6	45.3 ± 10.4	38.8 ± 6.5
Total mass (kg)	72.2 ± 18.3	68.2 ± 19	68.7 ± 9.0	80.9 ± 21.1
FM (kg)	25.4 ± 11.5	19.1 ± 10.4	30.8 ± 9.6	30.7 ± 10.4
BMC (kg)	2.6 ± 0.6	2.8 ± 0.6	2.3 ± 0.3	2.6 ± 0.6
DXA FFM (kg)	46.8 ± 10.6	49.1 ± 9.7	37.9 ± 3.2	50.2 ± 12.3
pFFM (kg)	47.2 ± 5.4	46.1 ± 5.4	46.3 ± 2.7	49.5 ± 6.7
LM (kg)	44.2 ± 10.0	46.3 ± 9.2	35.6 ± 3.0	47.6 ± 11.8
NLI	10 Tetraplegic (38%)	8 Tetraplegic (67%) C5–C7	2 Tetraplegic (33%) C4–C6	
	16 Paraplegic (62%)	4 Paraplegic (33%) T4–T11	4 Paraplegic (67%) T1–T12	8 Paraplegic (100%) T7–T12
TSI (years)	11.6 ± 10.3	12.8 ± 12.6	11.5 ± 9.9	10.0 ± 7.3
AIS	17 A (65%)	7 A (58%)	3 A (50%)	7 A (87.5%)
	4 B (15%)	2 B (17%)	2 B (33%)	1 C (12.5%)
	5 C (19%)	3 C (25%)	1 C (17%)	
BMR (kcal/day)	1452 ± 258	1436 ± 213	1290 ± 114	1597 ± 333

*Note*: Data are expressed as mean ± SD or percentage. One‐way ANOVA indicated that there were no differences (*p* = 0.08) in BMR among the three sub‐groups; IM, innervated males; DM, denervated males; IF, innervated females.

Abbreviations: AIS, American Spinal Injury Association Impairment Scale; BMC, bone mineral content; BMI, body mass index; BMR, basal metabolic rate (kcal/day); cm, centimeters; DXA, dual‐energy x‐ray absorptiometry; FFM, fat‐free mass; FM, fat mass; kg, kilograms; kg/m^2^, kilograms per meter squared; LM, lean mass; NLI, neurologic level of injury; pFFM, predicted fat‐free mass; TSI, time since injury.

### 2.2 Indirect calorimetry

Participants were instructed to refrain from exercise, caffeine, nicotine, and alcohol at least 12 h prior to testing period in accordance with minimal criteria for best practice BMR guidelines (Nevin, Mayr, et al., 2016). Participants were escorted to either a clinical research center or local hotel to spend the night before measuring BMR in the hotel room prior to getting out of bed. Following a 12‐h overnight fast, participants were woken up gently at ~6:30 a.m. to undergo a BMR assessment. All measurements were completed in a dark, quiet, thermoneutral environment (temperature between 20°C and 25°C). BMR was measured using a portable metabolic system (COSMED K4b^2^, Rome, Italy), and the unit was calibrated according to manufacturer guidelines (McLaughlin et al., 2001). Following calibration, a canopy was placed over the participant's head while they were in a supine position. Oxygen consumption was measured with continuous breath‐by‐breath measurements for over a 20‐min period. Participants were monitored to ensure they did not fall asleep during data collection and that no interruptions occurred. Gas exchange values collected during the initial 5‐min period were discarded, and BMR (kcal/day) was averaged for the remaining 15 min. Energy expenditure was determined using the Weir equation (JDV, 1949). If respiratory exchange ratio (RER; carbon dioxide production/oxygen used) values were <0.70 or >1.00, participants were excluded from analysis, as such values are indicative of an inaccurate gas measurements or protocol violation (Compher et al., 2006).

### 2.3 SCI body weight and height

Prior to measuring body weight and height, participants were instructed to void their bladder. Body mass (kg) was acquired using a digital wheelchair scale (Tanita PW‐630 U, IL), and participants body mass was calculated by subtracting the weight of the wheelchair from the combined weight of the participant and the wheelchair. Each participants height was measured in a supine position using two wooden boards, one placed at the tip of the head and the other placed at the sole of the foot. Following proper positioning, a Holtain–Kahn height caliper was used to measure the distance between the two wooden boards to the nearest 0.1 cm. A standard inflexible measuring tape (MFG, Lufkin, Executive Diameter Pocket Tape Measure) was used to obtain circumference measurements. Measurements were reported to the nearest 0.1 cm. If there was a difference greater than 0.5 cm in between readings, measurements were repeated until three measurements were within 0.5 cm range of each other, and the average of these measurements was used.

### 2.4 Anthropometrics

Participants were instructed to take a deep breath, and following exhalation of the preceding deep breath, the measurement was taken. Waist circumference was measured at the midpoint between the iliac crests and inferior margin of the last rib. Abdominal circumference was measured at the level of the umbilicus. Hip circumference was measured around the widest part of the greater trochanters. Sagittal and transverse abdominal diameters were also measured at the level of the umbilicus using a Holtain–Kahn abdominal caliper. Thigh circumference of the right lower extremity was measured at the midpoint between the anterior superior iliac spine and the superior border of the patella. Calf circumference of the right leg was measured at the widest point. Circumferential measurements were all taken in a supine position, except for the calf, which was measured with participants sitting in their wheelchair (Nightingale & Gorgey, 2018).

### 2.5 Dual energy x‐ray absorptiometry (DXA)

Total body DXA scans were performed using a GE lunar iDXA or Prodigy scanner (Lunar Inc., Madison, WI, USA) bone densitometer. DXA scans were used to quantify regional and total FM, FFM, %FM, LBM, and bone mineral content (BMC). Participants were transferred onto the DXA scanner and rested for 20 min in a flat supine position to account for potential fluid shift prior to the scan. All scans were performed and analyzed by a DXA‐trained researcher using enCORE GE Healthcare software. Whole‐body FFM was calculated by taking the sum of total body LM and BMC. In addition, whole‐body FFM was also predicted using the equation by Gorgey et al. (Gorgey et al., 2012)

$$\mathrm{FFM}=0.288\times \text{body weight}\;\left(\mathrm{kg}\right)+26.3$$

The following equation was used to predict whole‐body FFM to assess if BMR could be derived in the absence of a direct DXA FFM measurement.

### 2.6 BMR prediction equations

A total of 12 prediction equations were used to estimate BMR in this study (Table 1). SCI‐specific equations developed by Chun et al. and Nightingale & Gorgey both included FFM (Chun et al., 2017; Nightingale & Gorgey, 2018). Four different equation models were developed by Nightingale & Gorgey, in which Models 1 through 3 included either FFM alone or FFM plus anthropometrics. The Model 4 equation only included anthropometric parameters (height, weight, and transverse abdominal diameter). AB‐specific equations by Cunningham et al. and Owen et al. included body composition parameters using FFM, while the equation by Nelson et al. incorporated both (FFM & FM) with two different equations dependent on sex. (Cunningham, 1991; Nelson et al., 1992; Owen et al., 1987). The four AB‐specific equations developed by Harris & Benedict, Mifflin et al., Schofield et al., and Henry incorporated the following parameters (weight, height, and age) and also included two different equations dependent on sex (male or female) (Harris & Benedict, 1918; Henry, 2005; Mifflin et al., 1990; Schofield, 1985). The Schofield et al. and Henry equations used three separate equations depending on the participants' age group (age 18–30, 30–60, and >60 yrs.) to predict BMR from weight. Additionally, equations by Owen et al., Nelson et al., and Mifflin et al. measured RMR instead of BMR (Mifflin et al., 1990; Nelson et al., 1992; Owen et al., 1987).

### 2.7 Statistical analysis

The normality of the data was checked using Shapiro–Wilk Tests (*p* < 0.05), and if required, the data were log‐transformed prior to any statistical analysis. A paired sample *t*‐test was conducted to compare the measured BMR for individuals in each group and the results of each individual prediction equation. Independent *t*‐tests were used to examine significant mean difference in physical characteristics, body composition, anthropometrics, and predicted BMR for matched innervated females and males. Evaluation of differences in measured BMR between innervated and denervated males and innervated females was conducted using a one‐way analysis of variance (ANOVA) with Tukey's post hoc when significance was *p* < 0.05. Bland–Altman plots were applied to determine mean bias and identify the limit of agreement between measured and predicted BMR measurements for all prediction equations. In addition, univariate linear regressions were performed to derive the variance for innervated males with SCI only because of the small sample size in the denervated males [*n* = 8] and innervated females [*n* = 6]. The root mean square error (RMSE) was calculated to assess the predictive performance for each individual BMR prediction equation. A lower RMSE indicates a better performance of the BMR equation in estimating the actual BMR. Differences in predicted and measured BMR were calculated for each participant both as the bias in kcal/day (predicted BMR – measured BMR), and as a percentage difference between predicted BMR and measured BMR values. Mean absolute percent error was calculated by:

$$\begin{array}{c} \text{MAPE}\;\left(\%\right)\\ =\left[\left(\text{Predicted}\;\mathrm{BMR}–\text{measured}\;\mathrm{BMR}\right)/\text{measured}\;\mathrm{BMR}\right]\times 100 \end{array}$$

An accurate predicted BMR value was defined as one that fell within ±10% of the value obtained through indirect calorimetry (i.e., measured BMR), while overpredictions were considered to be ≥10% and underpredictions were ≤−10% (Frankenfield et al., 2003; JDV, 1949). Statistical analyses were performed using SPSS (SPSS statistics version 28.0.0.0, IBMR Corp, Armonk, USA). Bland–Altman plots were created using MedCalc (version 20.1.18, MedCalc Software Ltd., Ostend, Belgium). Physical characteristics and results are expressed as mean ± standard deviation. Statistical significance was set at an alpha level of *p* < 0.05.

## 3 RESULTS

### 3.1 Physical characteristics of participants

Participants' physical characteristics and demographics are presented in Table 2. A total of 26 participants were included in the analysis, which included 12 innervated males, six innervated females, and eight denervated males. Participants with tetraplegia represent sixty‐one percent (61%) of the sample size, while thirty‐nine percent (39%) were paraplegic.

### 3.2 Predicted BMR in males with innervated SCI

**FIGURE 1:**
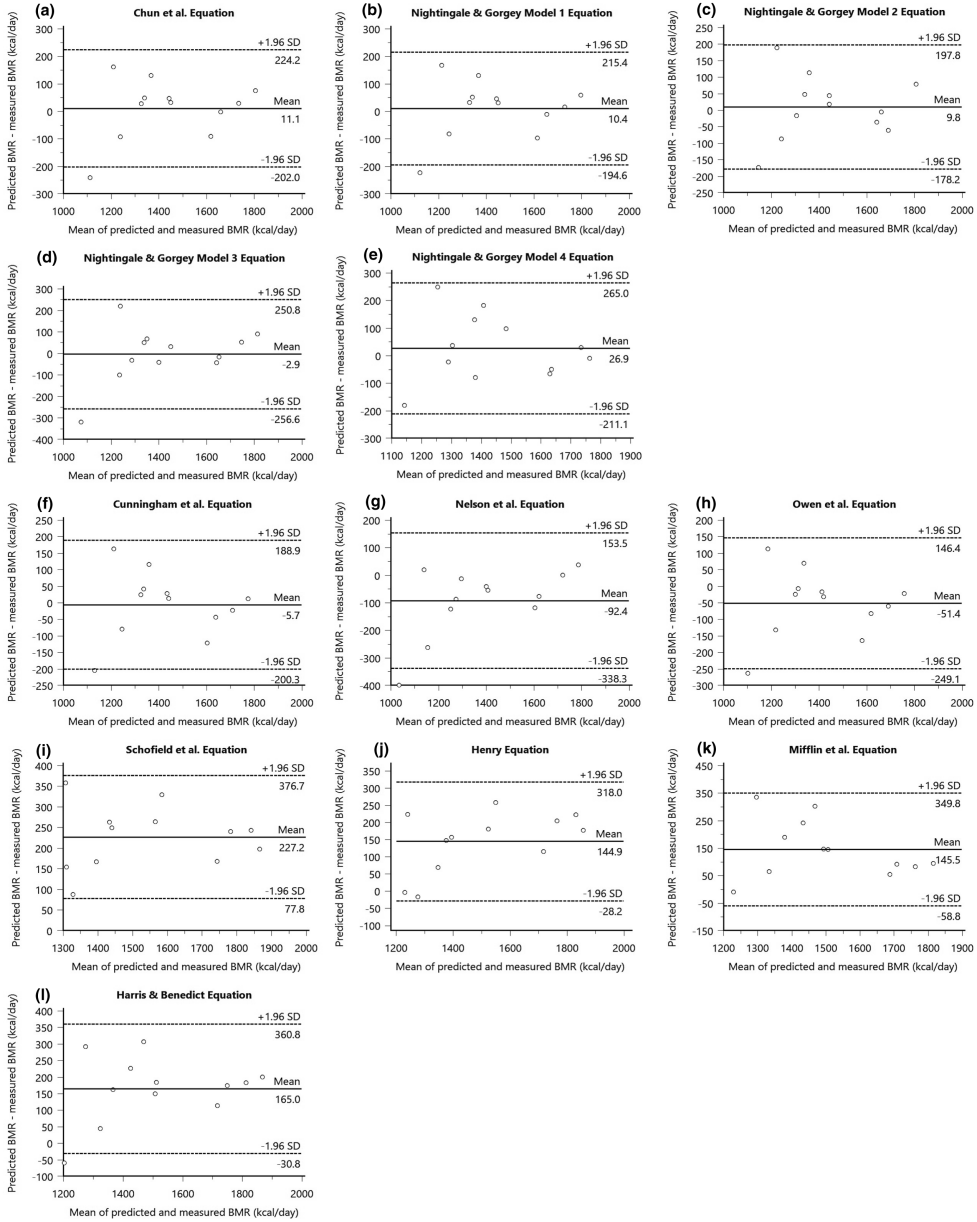
Bland–Altman plots depicting mean bias (solid line) and 95% LoA (dashed lines) of predicted BMR relative to measured BMR measured by indirect calorimetry for innervated males for SCI‐specific equations (a) Chun et al. equation, (b) Nightingale & Gorgey Model 1 equation, (c) Nightingale & Gorgey Model 2 equation, (d) Nightingale & Gorgey Model 3 equation, (e) Nightingale & Gorgey Model 4 equation, and AB‐specific equations, (f) Cunningham et al. equation, (g) Nelson et al. equation, (h) Owen et al. equation, (i) Schofield et al. equation, (j) Henry equation, (k) Mifflin et al. equation, (l) Harris & Benedict equation. Bias represents predicted‐measured BMR.

**FIGURE 2:**
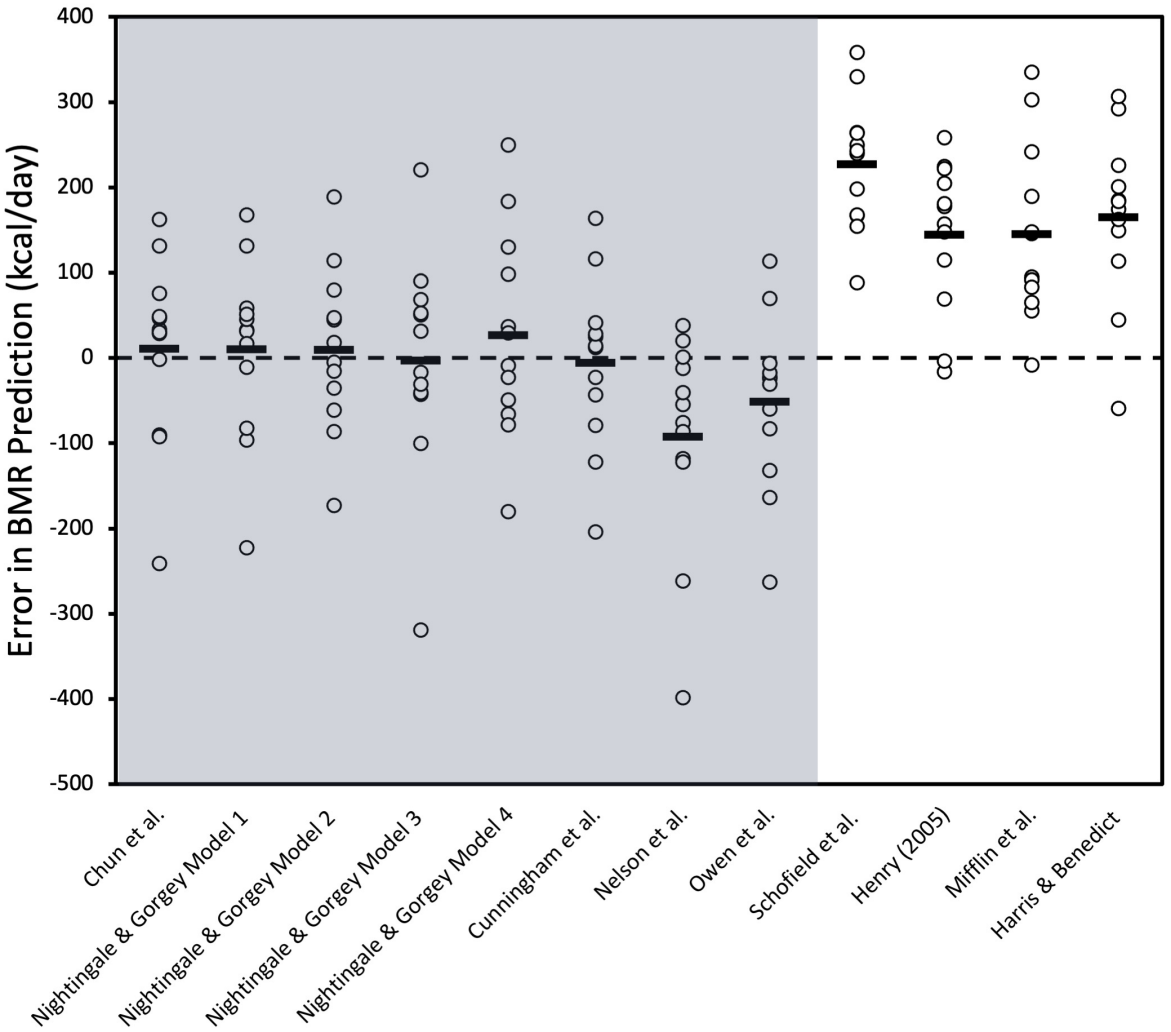
Scatterplot displaying BMR prediction error for each SCI‐specific and AB‐specific prediction equations for innervated males. Mean error for each equation is displayed as a thick black bar, with individual data points also shown (open circles). The highlighted areas (gray) are for the SCI‐specific equations and also AB‐specific equations that use FFM to predict BMR. The dashed line represents zero prediction error.

**FIGURE 3:**
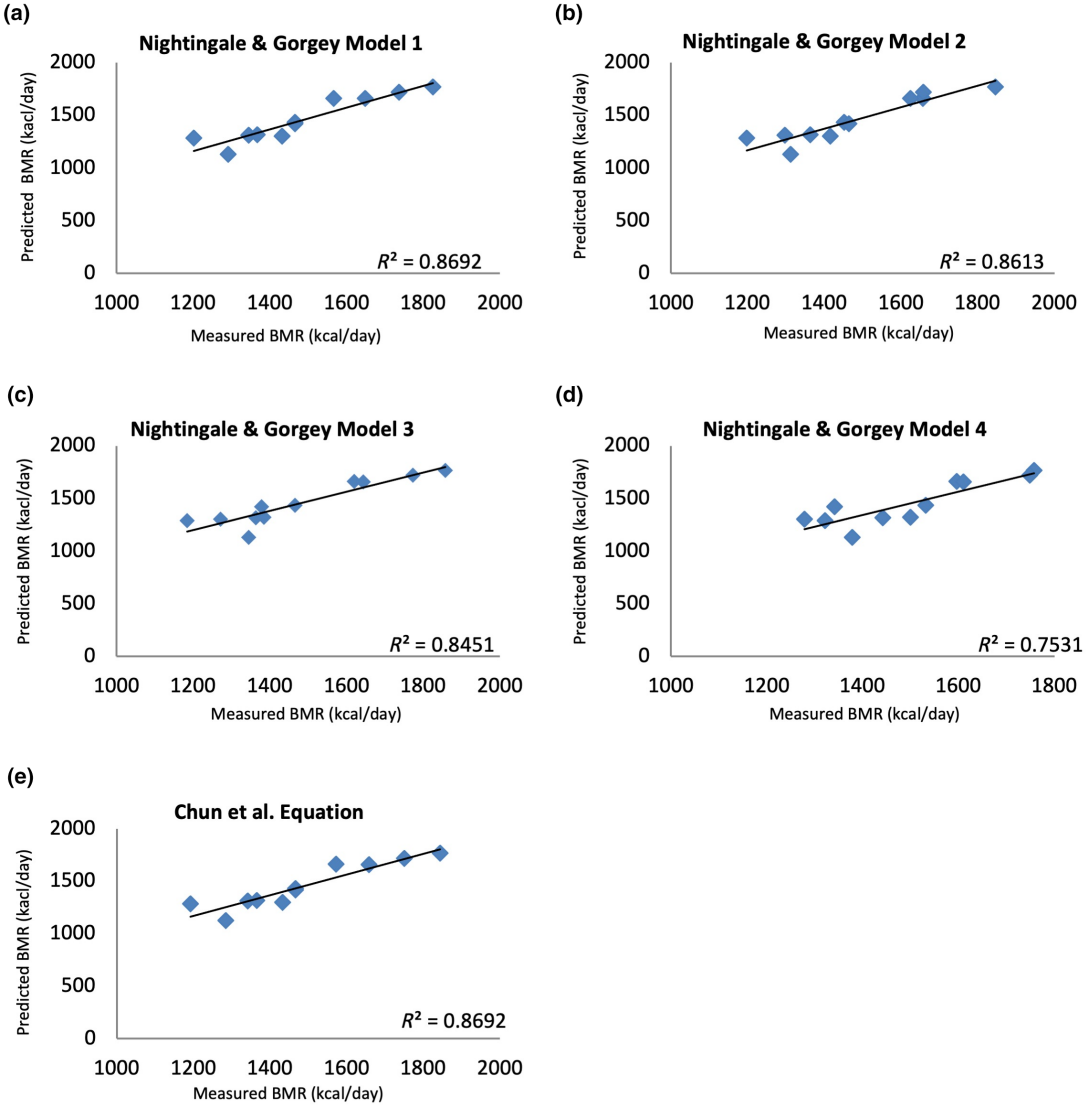
Linear regression analysis highlighting the relationship between predicted and measured BMR in innervated males with SCI using SCI‐specific equations (a) Nightingale & Gorgey Model 1 equation, (b) Nightingale & Gorgey Model 2 equation, (c) Nightingale & Gorgey Model 3 equation, (d) Nightingale & Gorgey Model 4 equation, (e) Chun et al. equation.

Table 3 summarizes the mean and mean differences between the measured BMR and predicted BMR for the group of innervated males with SCI. The results of the Bland–Altman plot analyses for SCI‐specific and AB‐specific prediction equations presented in Figure 1 and the variability in error for these equations displayed in Figure 2. Linear regression analyses for the innervated SCI males were displayed in Figure 3. The measured BMR for innervated males was 1436 ± 213 kcal/day. No significant differences were found between measured and predicted BMR using SCI‐specific equations by Chun et al. (*p* = 0.73), Model 1 (*p* = 0.74), Model 2 (*p* = 0.73), Model 3 (*p* = 0.94), and Model 4 (*p* = 0.46) by Nightingale & Gorgey, with Chun et al., Model 1, and Model 4 achieving 75% accuracy, and Models 2 and 3 reaching 83% accuracy, where Model 2 had the lowest RMSE value of 92 (Table 4) (Chun et al., 2017; Nightingale & Gorgey, 2018). The 95% LoA for individual differences in BMR predictions ranged from 224 to −202 kcal/day for Chun et al. and 215 to −195 kcal/day for Model 1, 198 to −178 kcal/day for Model 2, and a slightly wider range for Models 3 and 4, from 251 to −257 kcal/day and 265 to −211 kcal/day, respectively (Figure 1a–e).

**TABLE 3:** Agreement of prediction equations with measured basal metabolic rate in innervated males with SCI.

Innervated males (*n* = 12)						
	Mean ± SD (kcal/day)	Mean difference	ULOA	LLOA	MAPE (%)	d, 95% CI
Measured‐BMR	1436 ± 213					
Chun et al.	1447 ± 238	11 ± 109	224	−202	0.8%	−0.102 (−0.667, 0.468)
Nightingale & Gorgey Model 1	1447 ± 228	10 ± 105	215	−195	0.9%	−0.099 (−0.664, 0.470)
Nightingale & Gorgey Model 2	1446 ± 222	10 ± 96	198	−178	0.8%	−0.102 (−0.667, 0.468)
Nightingale & Gorgey Model 3	1433 ± 261	−3 ± 129	251	−257	−0.3%	−0.022 (−0.544, 0.588)
Nightingale & Gorgey Model 4	1463 ± 204	27 ± 121	265	−211	26.9%	−0.222 (−0.790, 0.356)
Cunningham et al.	1431 ± 210	−6 ± 99	199	−200	−0.1%	0.058 (−0.510, 0.623)
Nelson et al.	1344 ± 285^a^	−92 ± 125	154	−338	−7.0%	0.737 (0.081, 1.37)
Owen et al.	1385 ± 217	−51 ± 101	146	−249	−3.4%	0.509 (−0.105, 1.10)
Schofield et al.	1664 ± 217^a^	227 ± 76	377	78	16.2%	−2.98 (−4.32, −1.62)
Henry	1581 ± 258^a^	145 ± 88	318	−28	10.0%	−1.64 (−2.51, −0.744)
Mifflin et al.	1582 ± 185^a^	146 ± 104	350	−59	10.8%	−1.40 (−2.19, −0.573)
Harris & Benedict	1601 ± 237^a^	165 ± 100	361	−31	11.7%	−1.65 (−2.52, −0.752)

*Note*: MAPE (%) = [(Predicted BMR – measured BMR)/measured BMR] × 100. Data are expressed as mean ± SD or percentage.

Abbreviations: 95% CI, 95% confidence interval; d, Cohen's d; LLOA, lower limits of agreement; MAPE, mean absolute percent error (%); ULOA, upper limits of agreement.

^a^ Significantly different between predicted and measured BMR (kcal/day) via indirect calorimetry (*p* < 0.05).

**TABLE 4:** Accuracy, overprediction, and underprediction for each prediction equation compared to BMR measured through indirect calorimetry.

SCI‐specific equations						AB‐specific equations						
Equation	Chun et al.	Model 1	Model 2	Model 3	Model 4	Cunningham et al.	Nelson et al.	Owen et al.	Schofield et al.	Henry	Mifflin et al.	Harris & Benedict
Innervated males (*n* = 12)												
Accurate (%)	75	75	83	83	75	83	83	75	8	33	50	25
Overpredicted (%)	17	16	8	8	17	8	0	8	92	67	50	75
Underpredicted (%)	8	8	8	8	8	8	17	17	0	0	0	0
RMSE	105	101	92	124	119	95	152	109	239	168	176	191
Innervated females (*n* = 6)												
Accurate (%)	50	50	83	50	83	67	17	17	50	50	83	50
Overpredicted (%)	50	50	17	17	0	0	0	0	50	50	17	50
Underpredicted (%)	0	0	0	33	17	33	83	83	0	0	0	0
RMSE	140	132	173	229	113	128	200	224	155	124	72	137
Denervated males (*n* = 8)												
Accurate (%)	75	75	88	63	38	63	38	50	38	38	63	38
Overpredicted (%)	0	0	0	0	13	0	0	0	63	63	38	63
Underpredicted (%)	25	25	13	38	50	38	63	50	0	0	0	0
RMSE	155	158	130	153	210	177	205	214	284	234	164	217

*Note*: For each equation, data are expressed as percent of total sample. Each (accurate, overpredicted, and underpredicted) row sums to 100%. Accurately predicted basal metabolic rate (BMR) values fell within ±10% of the value obtained from indirect calorimetry. Overpredicted basal metabolic rate values were ≥10% of the value obtained from indirect calorimetry. Underpredicted basal metabolic rate values were ≤−10% of the value obtained from indirect calorimetry. RMSE (kcal/day), root mean square error. Models 1–4 are SCI‐specific equations by Nightingale & Gorgey (Nightingale & Gorgey, 2018).

AB‐specific prediction equations by Cunningham et al. and Owen et al. showed no significant differences from the measured BMR (*p* = 0.85 and *p* = 0.11, respectively), with accuracies of 83% and 75% for innervated males (Cunningham, 1991; Owen et al., 1987). In contrast, the Nelson et al. (Nelson et al., 1992) equation, while significantly different (*p* = 0.03), still achieved an 83% accuracy rate (Tables 3 and 4). The 95% LoA for individual differences in BMR predictions were 199 to −200 kcal/day for Cunningham et al., 146 to −249 kcal/day for Owen et al., and 154 to −338 kcal/day for Nelson et al. (Figure 1f–h). Additionally, the Cunningham et al. equation had a lower RMSE of 95 kcal/day, indicative of its predictive precision (Table 4).

Moreover, AB‐specific prediction equations by Schofield et al., Henry, Mifflin et al., and Harris & Benedict significantly overpredicted BMR for innervated males with SCI (*p* < 0.001), with Schofield et al. overpredicting for 92% of the sample, Henry for 67%, Mifflin et al. being 50% overpredictive and 50% accurate, and Harris & Benedict overpredicting for 75% and accurate for 25% of the sample (Tables 3 and 4) (Harris & Benedict, 1918; Henry, 2005; Mifflin et al., 1990; Schofield, 1985). The 95% LoA for individual differences in BMR predictions were 337 to 78 kcal/day for Schofield et al., 318 to −28 kcal/day for Henry, 350 to −59 kcal/day for Mifflin et al., and 361 to −31 kcal/day for Harris & Benedict, with Schofield et al. having the highest RMSE at 239 kcal/day, reflecting the variability in predictive performance. (Table 4) (Figure 1i–l).

### 3.3 Predicted BMR in males with Denervated SCI

**FIGURE 4:**
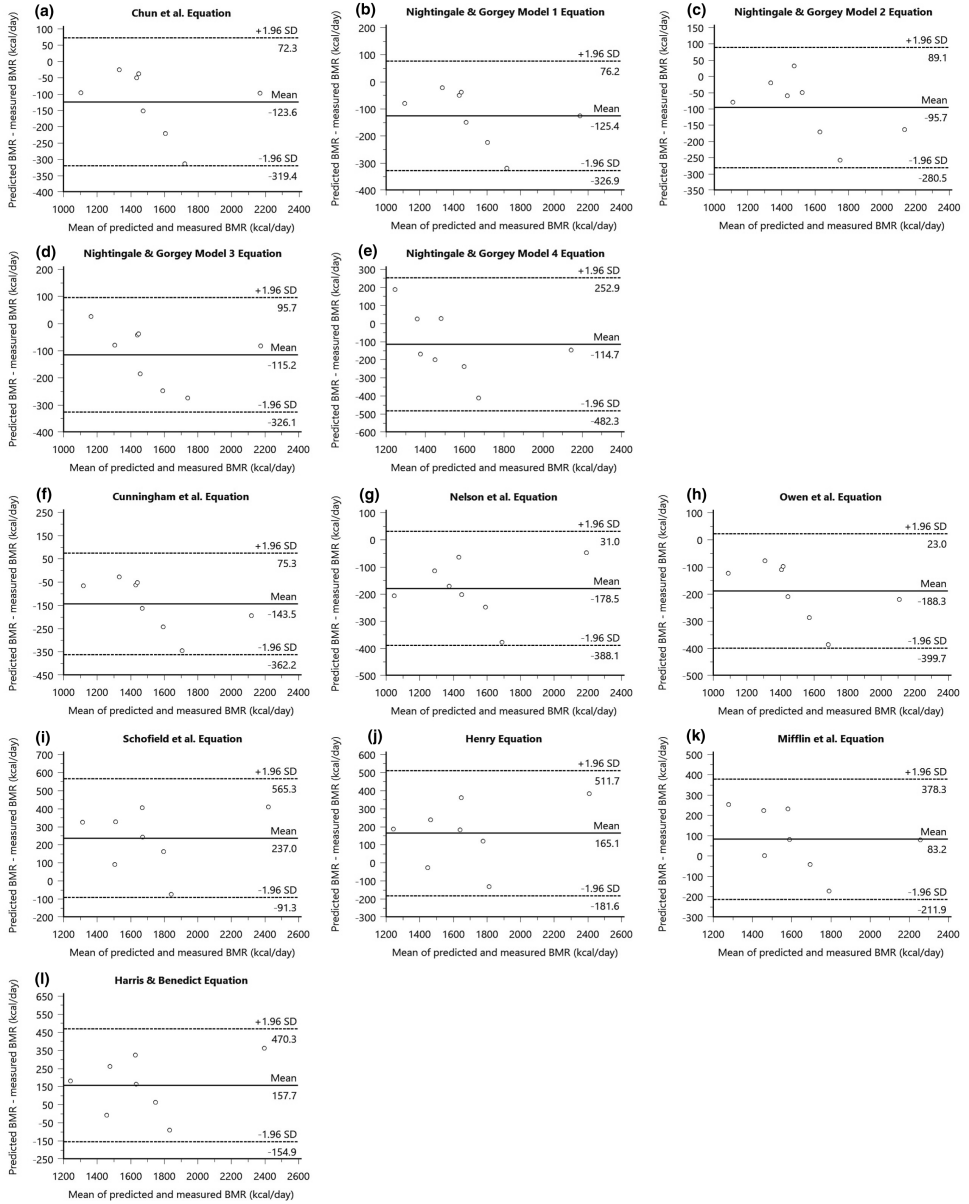
Bland–Altman plots depicting mean bias (solid line) and 95% LoA (dashed lines) of predicted BMR relative to measured BMR measured by indirect calorimetry for denervated males for SCI‐specific equations (a) Chun et al. equation, (b) Nightingale & Gorgey Model 1 equation, (c) Nightingale & Gorgey Model 2 equation, (d) Nightingale & Gorgey Model 3 equation, (e) Nightingale & Gorgey Model 4 equation, and AB‐specific equations, (f) Cunningham et al. equation, (g) Nelson et al. equation, (h) Owen et al. equation, (i) Schofield et al. equation, (j) Henry equation, (k) Mifflin et al. equation, (l) Harris & Benedict equation. Bias represents predicted‐measured BMR.

**FIGURE 5:**
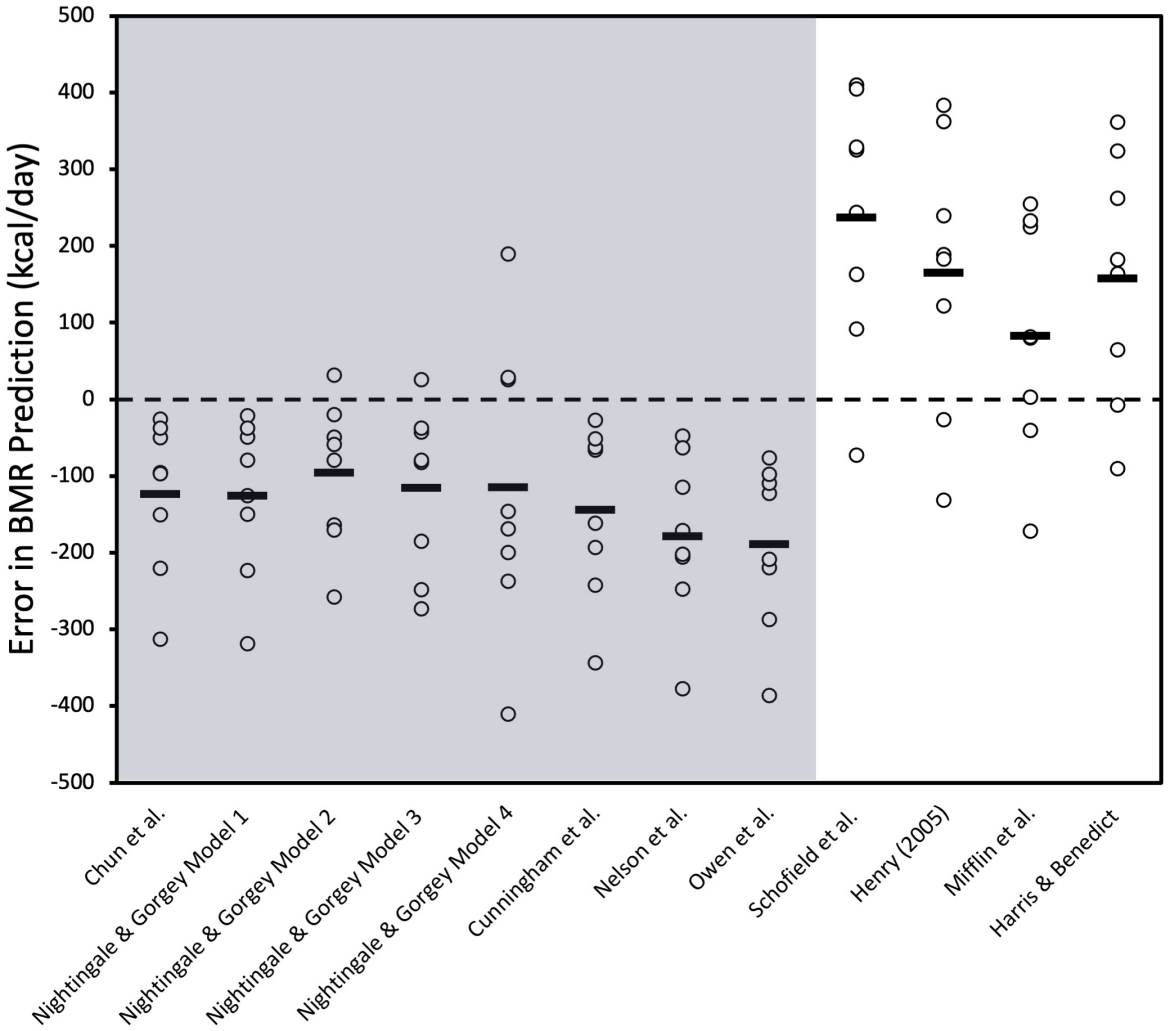
Scatterplot displaying BMR prediction error for each SCI‐specific and AB‐specific prediction equations for denervated males. Mean error for each equation is displayed as a thick black bar, with individual data points also shown (open circles). The highlighted areas (gray) are for the SCI‐specific equations and also AB‐specific equations that use FFM to predict BMR. The dashed line represents zero prediction error.

Table 5 shows that measured BMR for the group of denervated males with SCI was 1597 ± 333 kcal/day, with significant differences between measured and predicted BMR using the SCI‐specific equations by Chun et al. (*p* = 0.01), Model 1 (*p* = 0.01), Model 2 (*p* = 0.02), and Model 3 (*p* = 0.02) equations by Nightingale & Gorgey, while Model 4 showed no significant difference (*p* = 0.18) (Chun et al., 2017; Nightingale & Gorgey, 2018). At the individual level, accuracy ranged from 75% for equations by Chun et al. and Model 1, 88% for Model 2, 63% for Model 3, to the lowest at 38% for Model 4, which overpredicted BMR for half of the denervated sample (Table 4). The MAPE% for these equations showed a trend of underprediction, with −7.5% for both Chun et al. and Model 1, −5.5% for Model 2, −6.7% for Model 3, and a slightly lower error of −5.8% for Model 4 (Table 5) (Chun et al., 2017; Nightingale & Gorgey, 2018). The 95% LoA for individual differences in BMR predictions resulted in a substantial range, with Chun et al. ranging from 72 to −319 kcal/day, Model 1 from 76 to −327 kcal/day, Model 2 from 89 to −281 kcal/day, Model 3 from 96 to −326 kcal/day, and Model 4 from 253 to −482 kcal/day, reflecting a significant spread in the precision of these predictions (Figure 4a–e). RMSE values indicated Model 2 as the most precise with a value of 130 kcal/day, while Model 4 exhibited the least precision at 210 kcal/day (Table 4). Moreover, AB‐specific prediction equations developed by Cunningham et al. (*p* = 0.01), Nelson et al. (*p* = 0.002), and Owen et al. (*p* = 0.002) also significantly underpredicted BMR for the denervated male group. Further, the equation by Cunningham et al. underpredicted for 38% of the sample, Nelson et al. for 63% of sample, and Owen et al. underpredicted for 50% of the denervated sample. The RMSE values for the following AB‐specific equations ranged from 177 to 214, higher than those observed for SCI‐specific equations in addition to higher negative MAPE% values, reflecting less predictive performance in estimating BMR (Table 4 and Table 5). Moreover, 95% LoA for equations by Cunningham et al., Nelson et al., and Owen et al., were highly variable and less consistent across denervated individuals (Figure 4f–h). In contrast, AB prediction equations by Schofield et al. (*p* = 0.01), Henry (*p* = 0.03), and Harris & Benedict (*p* = 0.03) significantly overpredicted BMR for denervated males (Table 5). Additionally, the Schofield et al., Henry, and Harris & Benedict equations were all 38% accurate and overpredicted BMR by 63% for denervated males (Table 4). RMSE values for the following equations ranged from 164 to 284 kcal/day, while 95% LoA ranged from 565 to −182 kcal/day (Figure 4i–l). The variability in error for these equations are displayed in Figure 5.

**TABLE 5:** Agreement of prediction equations with measured basal metabolic rate in denervated males with SCI.

Denervated males (*n* = 8)						
	Mean ± SD (kcal/day)	Mean difference	ULOA	LLOA	MAPE (%)	d, 95% CI
IC‐BMR	1597 ± 333					
Chun et al.	1474 ± 302^a^	−124 ± 100	72	−319	−7.5%	1.23, (0.274, 2.153)
Nightingale & Gorgey Model 1	1472 ± 289^a^	−125 ± 103	76	−327	−7.5%	1.219, (0.263, 2.130)
Nightingale & Gorgey Model 2	1501 ± 279^a^	−96 ± 94	89	−281	−5.5%	1.015, (0.125, 1.859)
Nightingale & Gorgey Model 3	1482 ± 293^a^	−115 ± 108	96	−326	−6.7%	1.070, (0.164, 1.932)
Nightingale & Gorgey Model 4	1482 ± 249	−115 ± 188	253	−482	−5.8%	0.531, (−0.229, 1.260)
Cunningham et al.	1454 ± 266^a^	−143 ± 112	75	−362	−8.4%	1.285, (0.306, 2.219)
Nelson et al.	1419 ± 349^a^	−179 ± 107	31	−388	−11.5%	1.670, (0.547, 2.751)
Owen et al.	1409 ± 274^a^	−188 ± 108	23	−400	−11.4%	1.746, (0.593, 2.857)
Schofield et al.	1834 ± 352^a^	237 ± 168	565	−91	15.8%	−1.42, (−2.403, −0.392)
Henry	1762 ± 384^a^	165 ± 177	512	−182	10.8%	−0.933, (−1.754, −0.069)
Mifflin et al.	1680 ± 272	83 ± 151	378	−212	6.5%	−0.583, (−1.321, 0.189)
Harris & Benedict	1755 ± 373^a^	158 ± 159	470	−155	10.4%	−0.989, (1.826, −0.108)

*Note*: MAPE (%) = [(Predicted BMR – measured BMR)/measured BMR] × 100. Data are expressed as mean ± SD or percentage.

Abbreviations: 95% CI, 95% confidence interval; d, Cohen's d; LLOA, lower limits of agreement; MAPE, mean absolute percent error (%);ULOA, upper limits of agreement.

^a^ Significantly different between predicted and measured BMR (kcal/day) via indirect calorimetry (*p* < 0.05).

### 3.4 Predicted BMR in females with innervated SCI

**FIGURE 6:**
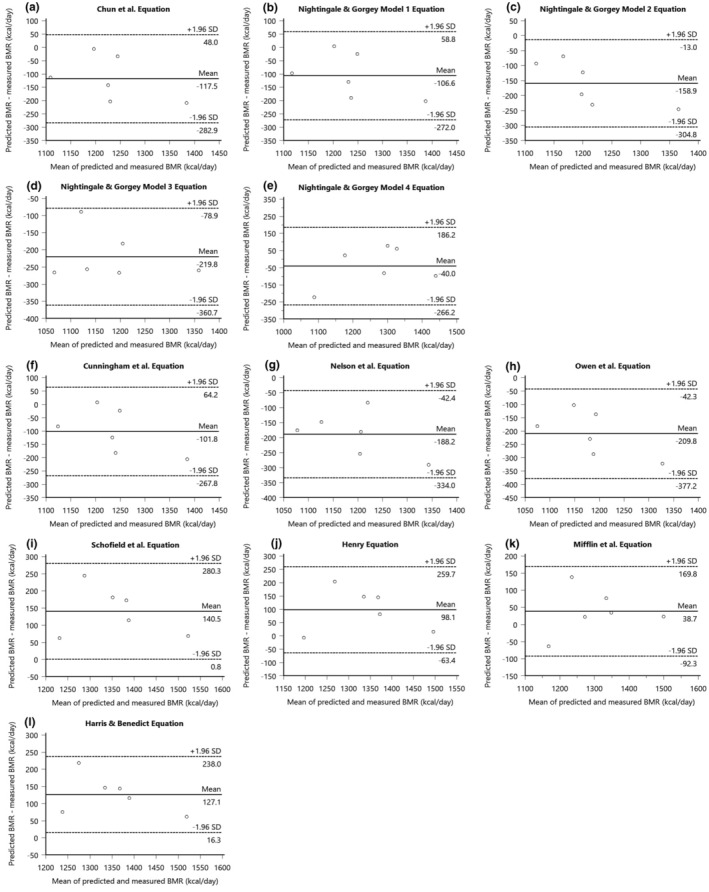
Bland–Altman plots depicting mean bias (solid line) and 95% LoA (dashed lines) of predicted BMR relative to measured BMR measured by indirect calorimetry for innervated females for SCI‐specific equations (a) Chun et al. equation, (b) Nightingale & Gorgey Model 1 equation, (c) Nightingale & Gorgey Model 2 equation, (d) Nightingale & Gorgey Model 3 equation, (e) Nightingale & Gorgey Model 4 equation, and AB‐specific equations, (f) Cunningham et al. equation, (g) Nelson et al. equation, (h) Owen et al. equation, (i) Schofield et al. equation, (j) Henry equation, (k) Mifflin et al. equation, (l) Harris & Benedict equation. Bias represents predicted‐measured BMR.

**FIGURE 7:**
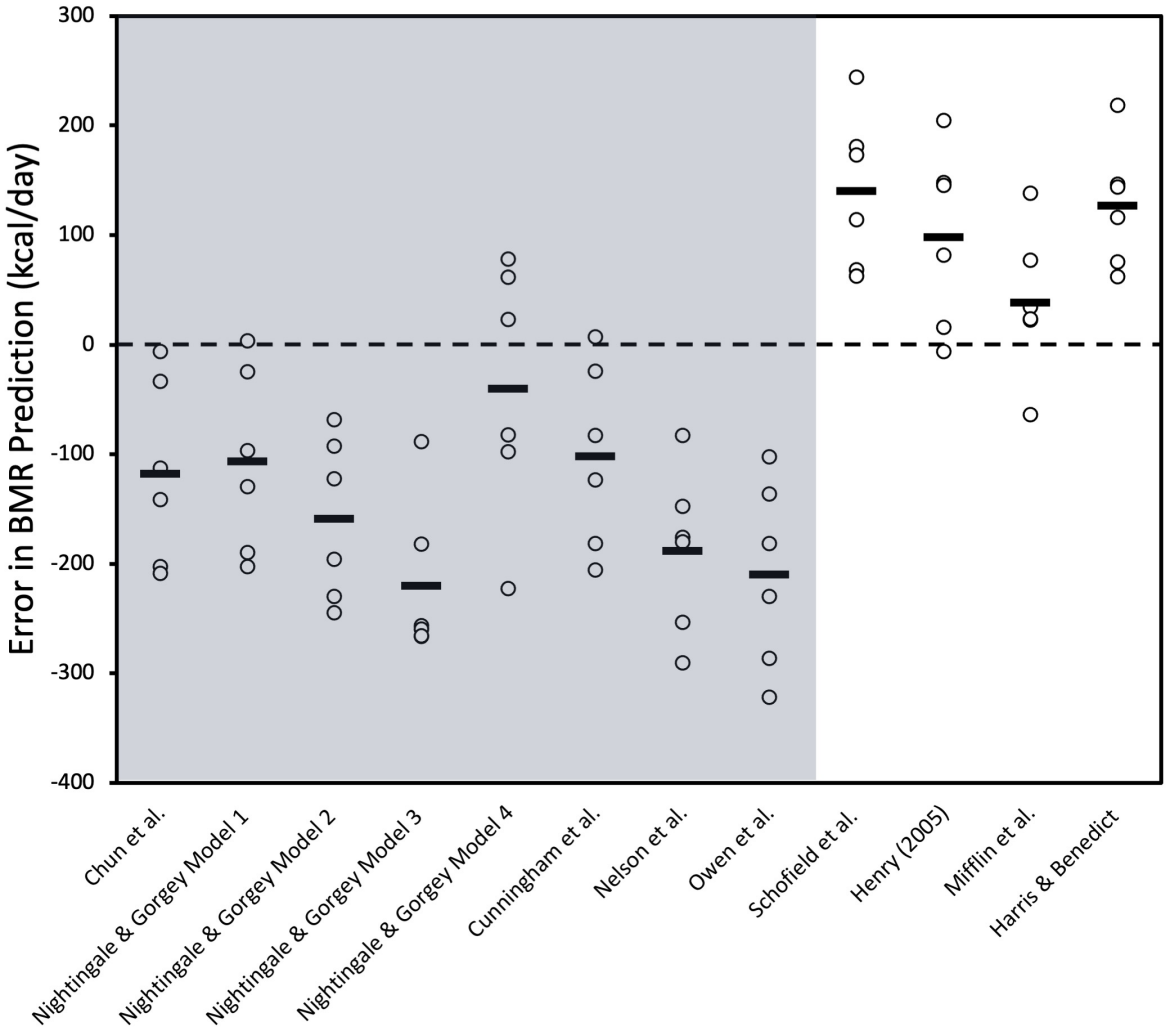
Scatterplot displaying BMR prediction error for each SCI‐specific and AB‐specific prediction equations for innervated females. Mean error for each equation is displayed as a thick black bar, with individual data points also shown (open circles). The highlighted areas (gray) are for the SCI‐specific equations and also AB‐specific equations that use FFM to predict BMR. The dashed line represents zero prediction error.

Table 6 shows that measured BMR for innervated females with SCI was 1290 ± 114 kcal/day, with significant differences between measured and predicted BMR for the SCI‐specific equations by Chun et al. (*p* = 0.02), Model 1 (*p* = 0.03), Model 2 (*p* = 0.003), and Model 3 (*p* = 0.001) by Nightingale & Gorgey, while Model 4 showed no significant difference (*p* = 0.44); Models 2 and 4 had 83% accuracy, and Chun et al., Model 1, and Model 3 had 50% accuracy in predicting BMR for the group of innervated females (Table 4) (Chun et al., 2017; Nightingale & Gorgey, 2018). The BMR prediction accuracy for innervated females with SCI varied across equations, with MAPE% ranging from −8.8% for Chun et al. to −16.9% for Model 3, with Model 4 presenting the least error at −3.1% (Table 6). The 95% limits of agreement further reflected this variability, with Chun et al. ranging from 48 to −283 kcal/day, Nightingale & Gorgey's Model 1 from 59 to −272 kcal/day, Model 2 from −13 to −305 kcal/day, Model 3 from −79 to −361 kcal/day, and Model 4 presenting a narrower range of 186 to −266 kcal/day (Figure 6a–e). The variability in error for SCI‐specific and AB‐specific prediction equations are displayed in Figure 7. Additionally, RMSE values complemented these findings, with Model 4 having the lowest RMSE at 113 kcal/day, suggesting its superior predictive precision, while Model 3 had the highest RMSE at 229 kcal/day, indicating greater prediction variability (Table 4). Moreover, AB‐specific prediction equations developed by Cunningham et al. (*p* = 0.03), with a MAPE% error of −7.6% and 67% accuracy, significantly underpredicted BMR for innervated females with SCI, as did the equations by Nelson et al. (*p* = 0.002) and Owen et al. (*p* = 0.002), which both had a MAPE% error of −14.4% and − 16.0%, respectively, and an accuracy of 17% (Table 6) (Cunningham, 1991; Henry, 2005; Nelson et al., 1992). The 95% limits of agreement for these equations were 64 to −268 kcal/day for Cunningham et al., −42 to −334 kcal/day for Nelson et al., and − 42 to −377 kcal/day for Owen et al., highlighting the range of BMR underprediction within this group (Figure 6f–h). Likewise, AB prediction equations by Schofield et al. (*p* = 0.01), Henry (*p* = 0.03), and Harris & Benedict (*p* = 0.003) significantly overpredicted BMR for innervated females, with MAPE% errors ranging from 7.9% to 11.2%, respectively (Table 6) (Compher et al., 2006; Mifflin et al., 1990; Schofield, 1985). Conversely, the Mifflin et al. equation did not show a significant difference from the measured BMR (*p* = 0.22), with a MAPE% of 3.1%, and was 83% accurate for the total female sample, overpredicting BMR by 17% (Tables 4 and 6) (Mifflin et al., 1990).

**TABLE 6:** Agreement of prediction equations with measured basal metabolic rate in innervated females with SCI.

Innervated females (*n* = 6)						
	Mean ± SD (kcal/day)	Mean difference	ULOA	LLOA	MAPE (%)	d, 95% CI
IC‐BMR	1290 ± 114					
Chun et al.	1173 ± 79^a^	−117 ± 84	48	−283	−8.8%	1.392, (0.204, 2.52)
Nightingale & Gorgey Model 1	1184 ± 76^a^	−107 ± 84	59	−272	−8.0%	1.263 (0.130, 2.33)
Nightingale & Gorgey Model 2	1131 ± 60^a^	−159 ± 74	−13	−305	−12.0%	2.135 (0.602, 3.62)
Nightingale & Gorgey Model 3	1070 ± 100^a^	−220 ± 72	−79	−361	−16.9%	3.057 (1.047,5.043)
Nightingale & Gorgey Model 4	1250 ± 153	−40 ± 115	186	−266	−3.1%	0.347 (−0.496, 1.158)
Cunningham et al.	1188 ± 70^a^	−102 ± 85	64	−268	−7.6%	1.202 (0.093, 2.250)
Nelson et al.	1102 ± 79^a^	−188 ± 74	−42	−334	−14.4%	2.531 (0.798, 4.228)
Owen et al.	1080 ± 64^a^	−210 ± 85	−42	−377	−16.0%	2.455 (0.761, 4.112)
Schofield et al.	1431 ± 96^a^	141 ± 71	280	+1	11.2%	−1.972 (−3.376, −0.519)
Henry	1388 ± 105^a^	98 ± 82	260	−63	7.9%	−1.191 (−2.235, −0.086)
Mifflin et al.	1329 ± 124	39 ± 67	170	−92	3.1%	−0.580, (−1.429, 0.317)
Harris & Benedict	1417 ± 90^a^	127 ± 57	238	16	10.1%	−2.248 (−3.79, −0.659)

*Note*: MAPE (%) = [(Predicted BMR – measured BMR)/measured BMR] × 100. Data are expressed as mean ± SD or percentage.

Abbreviations: 95% CI, 95% confidence interval; d, Cohen's d; LLOA, lower limits of agreement; MAPE, mean absolute percent error (%); ULOA, upper limits of agreement.

^a^ Significantly different between predicted and measured BMR (kcal/day) via indirect calorimetry (*p* < 0.05).

### 3.5 Differences in predicted BMR for matched male and females

Six innervated female participants were matched with six innervated male participants as closely as possible for NLI, TSI, and AIS classification (Table 7). The mean value of measured BMR for males was 1420 ± 250 kcal/day and 1290 ± 114 kcal/day for the female group (9.2%; *p* = 0.14).

**TABLE 7:** Physical and clinical baseline characteristics of matched innervated male and female participants.

Sex	Age	Weight (kg)	Height (cm)	BMI (kg/m^2^)	TSI (years)	NLI	AIS
Innervated Males (*n* = 6)							
M	51	83.2	181.3	25.3	1.75	T5	A
M	46	44.8	160.2	17.5	20	C6	A
M	44	53.5	183.0	16.0	13	C7	A
M	57	60.3	167.0	21.6	1.5	T11	C
M	48	95.2	183.2	28.4	17	T4	A
M	57	71.9	182.4	21.6	35	C5	A
Avg. ± SD	51 ± 6	68 ± 19	176 ± 10	22 ± 5	15 ± 13		
Innervated Females (*n* = 6)							
F	50	73.4	153.7	31.1	29	T3	A
F	51	69.4	164.1	25.8	13	C6	B
F	46	73.8	163.0	27.8	8	T1	A
F	24	72.2	171.3	24.6	4	T12	A
F	32	51.3	151.1	22.5	1	T4	A
F	55	76.7	166.7	27.6	14	C4‐C5	B
Avg. ± SD	43 ± 12	69 ± 9	162 ± 8	27 ± 3	12 ± 10		

*Note*: Average (Avg.) values are presented as mean ± standard deviation (SD).

Abbreviations: AIS, American Spinal Injury Association Impairment Scale.; BMI, body mass index; cm, centimeters; F, female; kg, kilograms; kg/m^2^, kilograms per meter squared; M, male; NLI, neurologic level of injury; TSI, time since injury.

Mean predicted BMR using the Chun et al. equation was significantly lower by 274 kcal/day for the female group compared to the male group (*p* = 0.026). Mean Predicted BMR using Model 1 and Model 2 equations by Nightingale & Gorgey was significantly lower by 263 kcal/day (*p* = 0.03) and 321 kcal/day (*p* = 0.01) for the female group. Mean predicted BMR using Model 3 was significantly lower by 346 kcal/day for females (*p* = 0.02); however, the Model 4 equation was 184 kcal/day lower for the female group (*p* = 0.08).

Predicted BMR for the Cunningham et al. equation was significantly lower by 242 kcal/day for the female group (*p* = 0.03). Similarly, the Owen et al. equation was significantly lower by 304 kcal/day for the female group (*p* = 0.01). The predicted BMR using the Nelson et al. equation was 238 kcal/day lower for the female group (*p* = 0.06). Moreover, equations by Schofield et al. and Mifflin et al. yielded a significantly lower predicted BMR for the female group (*p* = 0.03; *p* = 0.04). While the predicted BMR was not significantly different between both groups for equations by Henry and Harris & Benedict, both equations predicted a lower BMR for the female group (172 kcal/day; *p* = 0.09; 126 kcal/day; *p* = 0.17), respectively.

## 4 DISCUSSION

The main objectives of this study were to validate the existing BMR prediction equations in different subgroups of individuals with SCI, including innervated and denervated individuals, in an addition to examining the differences between predicted and measured BMR between females and males with SCI. This is the first study to evaluate the level of agreement between prediction equations for estimating BMR in SCI individuals with LMN in comparison to individuals with UMN injury. Our analysis demonstrated that prediction equations by Chun et al. and Nightingale & Gorgey (Models 1–4) accurately predicted BMR for the group of innervated males with no significant differences between the predicted and measured BMR (Chun et al., 2017; Nightingale & Gorgey, 2018). However, when we applied the SCI‐specific equations by Chun et al. and Nightingale & Gorgey (Models 1–3) to the group of denervated males, the predicted BMR was significantly lower from the measured BMR (Chun et al., 2017; Nightingale & Gorgey, 2018). Similarly, SCI‐specific equations by Chun et al. and Nightingale & Gorgey (Models 1–3) also significantly underpredicted BMR for the group of innervated females, while no significant difference was observed using the Model 4 equation by Nightingale & Gorgey, which incorporated only anthropometric and circumferential measurements (Farkas et al., 2019; Nightingale & Gorgey, 2018). We also demonstrated that AB‐specific equations by Schofield et al., Henry, and Harris & Benedict significantly overpredicted BMR in all three subgroups (Harris & Benedict, 1918; Henry, 2005; Schofield, 1985).

### 4.1 Innervated males with SCI

Previous reports have shown that FFM explains most of the variance in BMR (Bauman et al., 2004; Gorgey et al., 2010) suggesting that incorporating FFM in prediction equations more accurately predicts BMR instead of relying variables including height, weight, and age (Chun et al., 2017; Cunningham, 1991; Gorgey et al., 2010; Nightingale & Gorgey, 2018). SCI‐specific prediction equations by Chun et al. and Nightingale & Gorgey (Models 1–3), which incorporated FFM accurately predicted BMR in our group of innervated males, and MAPE% in the current study were nearly identical to the error difference reported by Chun et al. and Models (1–3) reported by Nightingale & Gorgey (Chun et al., 2017; Nightingale & Gorgey, 2018). However, using the Model 4 equation, which only incorporated anthropometric variables, we found the MAPE% higher by 20% than previously reported (Farkas et al., 2019; Nightingale & Gorgey, 2018). Moreover, the equations by Nightingale & Gorgey yielded an *r* ^2^ value that ranged from 0.57 to 0.77, with Model 3 presenting the highest value of 0.77 (Nightingale & Gorgey, 2018). Chun et al. reported an intra‐class correlation coefficient (ICC) value of 0.866; similarly, we yielded an *r* ^2^ value of 0.87.

Furthermore, we examined AB‐specific equations that also incorporated FFM, which included equations by Cunningham et al., Nelson et al., and Owen et al. (Cunningham, 1991;Nelson et al., 1992; Owen et al., 1987). The equation by Cunningham et al., had the second lowest RMSE value at the individual level (95 kcal/day) and accurately estimated BMR for 83% of the innervated group. The current findings were also similar to those reported by Chun and colleagues, as the authors reported a mean difference of (5.4 ± 118 kcal/day) when validating the Cunningham et al. equation (Farkas et al., 2019; Chun et al., 2017). We also found that the Nelson et al. equation, which incorporated FM in addition to FFM measurements significantly underpredicted BMR by −92 ± 125 kcal/day (Nelson et al., 1992). Similarly, Nightingale & Gorgey also showed that the Nelson et al. equation significantly underpredicted BMR by −82 ± 95 kcal/day (Nightingale & Gorgey, 2018). The Owen et al. equation underpredicted BMR less than the Nelson et al. (Nelson et al., 1992) equation with a mean difference of −51 ± 101 kcal/day and was found to be statistically equivalent to measured BMR for innervated males. Nelson et al. and Owen et al. measured RMR instead of BMR, which may have contributed to the difference in the predicted BMR (Nelson et al., 1992; Owen et al., 1987). In contrast, several prediction equations developed for AB populations rely strictly on parameters that do not consider differences in FFM, which include age, weight, and height, and rely on assumptions that previously have been shown to overestimate metabolic rate from 4% to 92% in individuals with SCI (Nevin, Steenson, et al., 2016). AB‐specific equations developed by Schofield et al., Henry, Mifflin et al., and Harris & Benedict all significantly overestimated BMR in the innervated sample (Harris & Benedict, 1918; Henry, 2005; Mifflin et al., 1990; Schofield, 1985). Moreover, Cox et al. reported that several energy equations, including the Harris & Benedict equation, overestimated BMR and RMR by as much as 46% of the SCI population (Cox et al., 1985). Similarly, Bauman et al. compared measured RMR values to predicted RMR values using the Harris & Benedict equation and reported a mean difference of 247 and 454 kcal/day, respectively, and showed an overestimation in RMR (Bauman et al., 2004; Harris & Benedict, 1918). Andersen et al. also reported that the commonly used Harris & Benedict and Mifflin et al. equation significantly overestimated RMR in the SCI population by 22% and 17% (Andersen et al., 2018). Overall, the current validation study underscores the significance of using SCI‐specific equations when predicting BMR requirements in different sub‐groups with SCI.

### 4.2 Denervated males with SCI

Lesions to the conus medullaris and cauda equina leads to irreversible denervation of LMN, which is accompanied by significant atrophy and flaccid paralysis affecting the lower extremities (Doherty et al., 2002). The relationship between level of injury and completeness has been reported to influence BMR, as higher‐level injuries result in a lower BMR due to greater muscle atrophy, decreased levels of activity, and diminished sympathetic drive (Kolpek et al., 1989; Mahadevan et al., 2008; Mollinger et al., 1985). In the current study, measured BMR for the denervated group was 1597 ± 333 kcal/day. Farkas et al. reported a mean measured BMR 1693 ± 329 kcal/day in five low paraplegic individuals using T9 as the demarcation point to account for individuals with a LMN (Farkas et al., 2020). In the present study, we showed that both SCI‐specific equations developed by Chun et al. and Nightingale & Gorgey (Models 1–3) significantly underpredicted BMR for the denervated male group. The additional circumferential and anthropometric measurements used in Model 2 may have contributed to predicting BMR better than predictive equations that only consider FFM. Additionally, the higher mean differences in predicted BMR for denervated males compared to innervated males using both SCI‐specific equations may be partly due to the fact that these equations were derived from a sample of chronic motor‐complete SCI with UMN injury. Likewise, the equation by Chun et al. was developed in East Asian motor‐complete SCI participants with a considerably lower mean FFM than participants from the Nightingale & Gorgey study (42.1 vs. 51.3 kg), while both studies did not include SCI participants with LMN (Chun et al., 2017; Nightingale & Gorgey, 2018). In a recent study by Alazzam et al. comparing muscle quality and body composition between matched innervated and denervated males, authors noted an approximately 10 kg difference in total LBM (Alazzam, Goldsmith, et al., 2023). Notably, differences in body composition (FFM, LM, & FM) between innervated and denervated SCI individuals are important to consider when using SCI‐specific equations that were derived from individuals with UMN SCI. Additionally, body composition differences warrant the need for additional research into the effects of LMN injury and metabolism, considering that individuals with LMN injury are estimated to account for nearly 25% of the SCI population (Alazzam, Goldsmith, et al., 2023; Chandrasekaran et al., 2020). In contrast, AB‐specific equations by Cunningham et al., Nelson et al., and Owen et al. were not as accurate compared to equations by Chun et al. and Nightingale & Gorgey, with LoA of agreement ranging 75 to −400 kcal/day (Chun et al., 2017; Cunningham, 1991; Nelson et al., 1992; Nightingale & Gorgey, 2018; Owen et al., 1987). Moreover, equations by Schofield et al., Henry, and Harris & Benedict significantly overpredicted BMR for the denervated group with 95% LoA ranging from 565 kcal/day to −182 kcal/day (Harris & Benedict, 1918; Henry, 2005; Schofield, 1985).

### 4.3 Innervated females with SCI

Previous literature has shown that in the AB population, males typically have a significantly higher RMR than females, which is primarily due to increased LBM in males compared to females (Arciero et al., 1993). However, following SCI, increases in FM and reductions in LBM are characterized according to sex, NLI, and completeness of injury (Farkas & Gater, 2018; Gorgey et al., 2018). The mean measured BMR for females was non‐significantly lower (1290 ± 114 kcal/day) in comparison to innervated males, where measured BMR was 1436 ± 213 kcal/day (Tables 3 and 6). Similarly, Gorgey et al. reported non‐significant differences in BMR by sex (males: 1421 kcal/day vs. females: 1367 kcal/day) (Gorgey et al., 2018). Additionally, Buchholz et al. examined differences in RMR by sex and showed a difference of 310 kcal/day between men and women with SCI, and greater LBM was noted in men (Buchholz et al., 2003). The current report highlighted the significance of customizing separate dietary plans based on differences in sex after SCI. It also underscores the need for more research to address the differences in metabolic requirements and caloric intake between males and females with SCI.

Considering the notable differences in BMR in males and females, we also examined the predictive accuracy of both SCI and AB‐specific equations. Using the Chun et al. equation for innervated females resulted in a significant underprediction in BMR with 95% LoA ranging from 48 to −283 kcal/day. The Chun et al. equation was developed on 50 individuals (38 males, 12 females); however, the authors did not report BMR values based on sex (Chun et al., 2017). Similarly, Models (1–3) by Nightingale & Gorgey also significantly underpredicted BMR with 95% LoA ranging from 59 to −361 kcal/day. In contrast, the Model 4 equation by Nightingale & Gorgey, which used only anthropometric measurements was statistically equivalent to the measured BMR (1250 ± 153 vs. 1290 ± 114). It is possible that the incorporation of transverse abdominal diameter in the Model 4 equation might have contributed to the precision of the equation, as previous reports have shown upper trunk visceral adipose tissue (VAT) adjusted to trunk FM and total body FM were significantly lower in women than men with SCI (Gorgey et al., 2018). The SCI‐specific equations by Nightingale & Gorgey (Nightingale & Gorgey, 2018) were developed in 30 males with chronic motor complete injury, which may explain the underprediction observed in Models (1–3). Moreover, AB‐specific equations by Schofield et al., Henry, and Harris & Benedict significantly overpredicted BMR for females with SCI (Compher et al., 2006; Mifflin et al., 1990; Schofield, 1985).

We also matched six females and six males for age, NLI, TSI, and AIS classification and found that measured BMR was 130 kcal/day lower for females, which confirms our previous findings (Gorgey et al., 2018). As previously noted, the SCI‐specific equations and AB‐specific equations that both incorporated FFM significantly underpredicted BMR for females. We observed that total body FFM (*p* = 0.026) and FM (*p* = 0.018) were significantly different between both groups, as females had on average 11.3 kg lower FFM and a higher FM of 12.6 kg. In addition, total body FFM was underpredicted by 8.4 kg for females using the Gorgey et al. (Gorgey et al., 2012) equation, which estimates FFM from body weight and was developed in a male SCI population. In the absence of expensive scanning equipment (i.e., DXA or indirect calorimetry), it is advisable to use the Model 4 equation to predict BMR in females with SCI. Differences in body composition between males and females may explain the underprediction of FFM‐based prediction equations. Moreover, equations by Schofield et al., Henry et al., and Harris & Benedict were all found to overpredict BMR for females.

Importantly, throughout SCI literature, several methodological differences in metabolic rate acquisition make it difficult to compare results from different groups. For instance, studies by Chun et al. (Chun et al., 2017) and Nightingale & Gorgey (Nightingale & Gorgey, 2018) measured BMR using a ventilated hood or canopy, while Ma et al. measured RMR using a mask (Ma et al., 2022), and in some cases, groups have alternatively relied on the use of a Douglas bag. For the measurement protocol, there is no consensus on measuring metabolic rate using indirect calorimetry, as several studies applied different rest periods before measuring RMR/BMR (Perret & Stoffel‐Kurt, 2011; Yilmaz et al., 2007), while Compher et al. recommended resting for at least 20 min before measurement (Compher et al., 2006). Such methodological differences may contribute to additional bias when comparing values across studies and lead to confusion when prescribing caloric needs for individuals with SCI. Using RMR to estimate caloric requirements may overestimate dietary needs, which could lead to inappropriate caloric intake recommendations that may increase the risk for obesity and secondary cardiometabolic complications (Alazzam, Alrubaye, et al., 2023).

## 5 STRENGTHS AND LIMITATIONS

A major strength of our study is successfully validating recently developed SCI‐specific prediction equations in different subgroups of SCI, specifically innervated and denervated individuals with SCI. Another strength is the standardized protocol in which all anthropometric, BMR, and body composition measurements were used. However, the current study was not powered because of the inherent limitation posed by the small sample sizes for females and individuals with LMN injury. Females with SCI constitute only 25% of the entire SCI population, and at the Department of Veteran Affairs, there is limited access to these specific sub‐populations with SCI. Consequently, the small sample size may have limited the statistical power needed to fully validate the accuracy of the prediction equations, especially in the subgroups of innervated females and denervated males.

Furthermore, the small sample size must be placed in context of the mean bias and limits of agreement between predicted and measured BMR values in the current sub‐groups. Likewise, the heterogeneity of impairment and completeness in our sample (paraplegic vs. tetraplegic) is important to note as some individuals may present differences in metabolic rate. In addition, spasticity characterized by involuntary and uncontrolled muscle spasms occurs in more than 70% of persons with SCI (Levi et al., 1995). During assessment of BMR, participants need to be in a rested, uninterrupted basal state; however, occurrences of spasticity or muscle spasms may have led to increased energy expenditure (Stoquart et al., 2005).

## 6 CONCLUSION

Common AB energy prediction equations including Schofield et al., Henry, Mifflin et al., and Harris & Benedict do not accurately estimate caloric needs within the innervated and denervated SCI population. The current findings from this study demonstrated that SCI‐specific and AB‐specific FFM prediction equations provided an accurate prediction of BMR for innervated males with SCI. In addition, the Model 4 (anthropometric/circumferential) equation by Nightingale and Gorgey was considerably accurate in predicting BMR in innervated females with SCI. However, future research is needed to develop SCI‐specific prediction equations for females that incorporate FFM and additional parameters. SCI‐specific equations by Chun et al. and Models (1–3) applied at the individual level accurately predicted BMR for denervated males with SCI. Moreover, caution is warranted as several equations underpredicted BMR at the group level, which further warrants additional research studies. It is highly recommended that researchers and clinicians acknowledge the overestimation of energy expenditure when using RMR instead of BMR to prescribe caloric requirements for individuals with SCI. Future studies with large sample sizes are needed to examine the influence of neurological level of injury, completeness of injury, and sex on BMR in different subgroups within SCI.

## CONFLICT OF INTEREST STATEMENT

We declare that none of the authors have any conflict of interest related to the work under consideration.

## ETHICS STATEMENT

We certify that all applicable institutional and governmental regulations concerning the ethical use of human volunteers were followed during the course of this research.

## Data Availability

The data was uploaded as supplementary material for review process only. After acceptance of the paper, the data will be available upon email communications with the corresponding author.
